# Low-Fishmeal Dietary Supplementation with Crayfish By-Product Protein Hydrolysate Affects Growth Performance, Appetite-Related Metabolic Signaling and Intestinal Microbiota of Pacific White Shrimp (*Litopenaeus vannamei*)

**DOI:** 10.3390/metabo16040221

**Published:** 2026-03-27

**Authors:** Lina Ren, Wanshan Gu, Huangbing Sun, Guoqiang Fan, Xiaojing Yang

**Affiliations:** 1 Key Laboratory of Animal Physiology & Biochemistry, College of Veterinary Medicine, Nanjing Agricultural University, Nanjing 210095, China; lina.ren@stu.njau.edu.cn (L.R.); guwanshan@njau.edu.cn (W.G.); sunhuangbing@stu.njau.edu.cn (H.S.); fan.gq@njau.edu.cn (G.F.); 2 MOE Joint International Research Laboratory of Animal Health and Food Safety, Nanjing Agricultural University, Nanjing 210095, China

**Keywords:** protein hydrolysate, crayfish by-products, appetite-related genes, intestinal microbiota, *Litopenaeus vannamei*

## Abstract

Background/Objectives: Low-fishmeal diets are widely adopted to improve sustainability in shrimp aquaculture, yet reduced palatability and metabolic stress frequently suppress feed intake and growth. We evaluated whether a crayfish (*Procambarus clarkii*) by-product protein hydrolysate (CBPH) could mitigate low-fishmeal-induced performance losses by modulating feeding-related metabolic signaling and gut microbiota features in Pacific white shrimp (*Litopenaeus vannamei*). Methods: In an 8-week feeding trial, 360 juveniles (initial body weight 0.46 g) were assigned to three diets (four replicates per diet): a commercial control (CON), a low-fishmeal diet (LFM), and LFM supplemented with 2% CBPH (CBPH). Growth, feed utilization, whole-body composition, hemolymph biochemical indices (TP, TG, GLU, AST, ALT), intestinal appetite-related gene expression (5-HTR, CART, CCK1R, D2-like, NPY), and intestinal microbiota profiles (full-length 16S rRNA sequencing, V1–V9, PacBio) were assessed. Results: Compared with the LFM group, CBPH supplementation increased feed intake and improved feed conversion, restoring final body weight and growth rates to levels comparable to CON. CBPH also alleviated low-fishmeal-associated metabolic stress, including reduced AST and ALT activities and lower glucose levels. The LFM diet induced upregulation of anorexigenic genes (5-HTR, CART, D2-like) and downregulation of NPY in the shrimp intestine, whereas CBPH supplementation reversed these transcriptional changes. In addition, microbiota richness indices (ACE and Chao1) were elevated by CBPH relative to LFM, accompanied by compositional shifts at the phylum and genus levels. Conclusions: CBPH effectively alleviated low-fishmeal-induced reductions in feeding and growth, accompanied by coordinated changes in feeding-related gene expression, systemic biochemical markers, and gut microbiota composition, supporting its potential as a functional ingredient to stabilize metabolic responses in low-fishmeal shrimp feeds.

## 1. Introduction

*Litopenaeus vannamei*, commonly known as Pacific white shrimp, is the dominant shrimp species in global aquaculture, attributed in part to its high growth efficiency, strong adaptability to diverse culture environments, desirable flesh quality, and broad tolerance to salinity fluctuations [1]. Over the past two decades, the global expansion of intensive and semi-intensive shrimp farming systems has driven a dramatic increase in the production of this species. In 2020, global Pacific white shrimp production surpassed 5.8 million tons, making up over half of all farmed shrimp and highlighting its importance in aquaculture and seafood supply chains [2]. As shrimp aquaculture continues to intensify, the optimization of feed formulations has become a central focus for improving production efficiency, reducing costs, and enhancing sustainability. Dietary protein is the most critical and costly nutrient in shrimp feeds, as shrimp have relatively high protein requirements to support rapid growth and efficient tissue accretion [3]. Traditionally, fishmeal has been considered the premium protein source in shrimp diets because of its well-balanced amino acid composition, superior digestibility, and outstanding palatability. In addition, fishmeal contains various bioactive compounds that stimulate feed intake and support optimal growth in shrimp. However, the long-term reliance on fishmeal has raised serious concerns regarding economic viability and environmental sustainability. Global fishmeal production is constrained by limited marine capture fisheries, while increasing demand from aquaculture has led to price volatility and supply insecurity [4]. These challenges have stimulated extensive research into low-fishmeal or fishmeal-free feeding strategies.

To reduce fishmeal inclusion, plant-derived protein ingredients such as soybean meals, soybean protein concentrate, rapeseed meal, and other oilseed by-products have been widely incorporated into shrimp diets [5,6]. Although these alternative protein sources can partially meet the nutritional requirements of shrimp, their extensive use is often associated with several drawbacks. Plant proteins generally contain anti-nutritional factors, imbalanced amino acid profiles, and lower levels of water-soluble attractants compared with fishmeal. As a result, high substitution levels of fishmeal with plant proteins frequently lead to reduced feed palatability, decreased voluntary feed intake, impaired nutrient utilization, and ultimately slower growth performance. Such negative effects have been documented in various aquatic species, including rainbow trout (*Oncorhynchus mykiss*) [7] and Pacific white shrimp [8,9]. Therefore, maintaining feed palatability and feeding motivation under low-fishmeal conditions has become a critical bottleneck for the successful implementation of sustainable shrimp feeds.

One promising approach to address this challenge is the incorporation of functional feed additives with strong attractant properties. Marine and aquatic processing by-products represent a large and underutilized reservoir of high-quality nutrients, particularly proteins and bioactive compounds [10]. Through enzymatic hydrolysis, these by-products can be converted into protein hydrolysates rich in low-molecular-weight peptides and free amino acids. Such compounds are highly soluble and can effectively stimulate chemoreceptors in shrimp, thereby enhancing feed detection, ingestion, and overall feeding behavior [11]. In addition to their attractant effects, protein hydrolysates may also exert physiological benefits, including improved digestion, enhanced immune responses, and modulation of intestinal microbiota. Among these, fish soluble pulp, made from fish by-products like skin, scales, bones, and organs, is high in protein and amino acids, and it has been widely used to enhance feed palatability and stimulate feeding behavior in Pacific white shrimp [12]. Studies have shown that squid-based products, like squid paste and visceral powder, are high in amino acids and can boost feeding and growth. For instance, squid extract has been found to increase feeding behavior and growth rates in Atlantic salmon [13], and squid viscera paste has also led to better feed intake and growth in Pacific white shrimp [14]. Recent research highlights a growing interest in developing novel protein hydrolysates from a wide range of aquatic animals and by-products, aiming to improve sustainability, health benefits, and feed efficiency in aquaculture.

The red swamp crayfish (*Procambarus clarkii*) is the largest proportion of commercially farmed freshwater crustaceans in China and has become an integral component of China’s aquatic economy because of its rapid growth and adaptability to the environment, and relatively high fecundity [15,16]. China stands as the world’s largest producer and consumer of crayfish, and in 2022, consumption reached 2.89 million tons [15]. This substantial demand resulted in the generation of more than 2 million tons of crayfish by-products, which account for approximately 70% of the total crayfish weight and include parts such as the head, claws, and shell [17,18]. Improper disposal of these residues not only represents a significant waste of resources but also poses potential environmental risks. Notably, the shell of the red swamp crayfish contains a high amount of protein, which makes it a useful ingredient as a feed additive for aquatic animals [19,20]. Recent studies have demonstrated that protein hydrolysates derived from crayfish shells possess favorable amino acid profiles and high digestibility. Dietary supplementation with crayfish shell protein hydrolysates has been shown to improve growth performance, muscle amino acid composition, and myofiber structure in zebrafish, indicating their potential as functional feed ingredients in aquaculture [21]. However, despite the abundance of crayfish by-products and their promising nutritional properties, information regarding their application in shrimp diets, particularly under low-fishmeal conditions, remains limited. The 2% inclusion level of CBPH was selected based on preliminary formulation trials, aiming to ensure diet feasibility and palatability under low-fishmeal conditions. Therefore, this study aimed to determine whether supplementation of a low-fishmeal diet with 2% crayfish by-product protein hydrolysate (CBPH) can mitigate low-fishmeal-associated performance losses by (i) improving feed intake and growth, (ii) modulating appetite-related gene expression and systemic biochemical biomarkers, and (iii) reshaping gut microbiota features associated with microbiota-related metabolic potential in Pacific white shrimp.

## 2. Materials and Methods

### 2.1. Experimental Diets

The ingredient composition and proximate analysis of the experimental diets are summarized in Table 1. Three practical diets with equal nitrogen and lipid content prepared for this experiment: a commercial diet was used as the control (CON), a low-fishmeal diet without CBPH supplementation served as the negative control (LFM) and the third diet consisted of the same low-fishmeal formulation supplemented with 2% CBPH (CBPH). Protein hydrolysate from by-products of red swamp crayfish (CBPH) was produced by enzymatic hydrolysis in our laboratory. All dry ingredients were ground, sieved through a 60-mesh screen, and uniformly mixed. Oils and liquid components were then added to form a consistent dough, which was processed into 1.0 mm diameter feeds using a laboratory pelletizer (South China University of Technology, Guangdong, China). The pellets were air-dried to approximately 10% moisture content, broken into suitable sizes, sealed in airtight containers, and stored at −20 °C prior to use.

### 2.2. Experimental Shrimp and Feeding

Juvenile Pacific white shrimp were obtained from a commercial hatchery (Yancheng, Jiangsu, China) and acclimated for 3 weeks while receiving a commercial shrimp feed. After acclimation, 360 healthy Pacific white shrimp (initial body weight 0.46 g) were randomly distributed into 12 cylindrical fiberglass tanks (300 L). Each diet was assigned to four replicate tanks. Shrimp were fed three times daily (08:00, 14:00, and 20:00) to apparent satiation for 8 weeks. Residual feed was siphoned one hour after feeding to assess feed utilization. Throughout the experiment, water temperature was maintained at 28–30 °C, salinity at 8–10‰, pH at 7.8–8.0, and dissolved oxygen levels above 7 mg L^−1^. Water quality parameters were measured twice weekly using commercial test kits (Sangpu, Beijing, China), and shrimp mortality was recorded on a daily basis.

### 2.3. Sample Collection

At the end of the feeding experiment, shrimp were deprived of feed for 24 h prior to sampling, after which they were counted and weighed. Five shrimp were randomly collected from each replicate and stored at −20 °C for subsequent whole-body composition analysis. In addition, four shrimp from each tank were individually measured for body length and body weight to determine the condition factor (CF). Subsequently, the hepatopancreas was carefully excised and weighed in order to assess the hepatopancreas somatic index (HSI). Hemolymph was collected from the pericardial cavity of ten shrimp per tank using sterile syringes, without adding anticoagulant. The samples from each tank were combined, homogenized, then centrifuged at 8000 rpm for 10 min at 4 °C. The supernatant (hemolymph serum) was obtained and stored at −80 °C for later biochemical analysis. Intestinal tissues were collected from six shrimp per tank, with two intestines pooled per sample, preserved in RNAlater (Thermo Fisher Scientific, Waltham, MA, USA), and stored at −80 °C prior to RNA extraction.

### 2.4. Chemical Analysis

Moisture, crude protein, crude lipid, and ash contents of the diets and shrimp tissues were determined following standard analytical procedures [22]. Crude protein was determined with a Kjeltec^TM^ 8400 system (FOSS, Alingsås, Sweden). Crude lipid content was assessed using an XT15 extractor (Ankom, Macedon, NY, USA). Ash content was measured by combusting samples in a muffle furnace at 550 °C for six hours. Hemolymph biochemical parameters, including total protein (TP), triglyceride (TG), glucose (GLU), aspartate aminotransferase (AST), and alanine aminotransferase (ALT), were quantified using an automatic biochemical analyzer (Hitachi High-Tech Corporation, Tokyo, Japan).

### 2.5. Gene Expression Analysis

Total RNA was obtained using TransZol Up Plus reagent (TransGen, Beijing, China) following the supplier’s protocol. RNA purity and concentrations were verified through agarose gel electrophoresis and spectrophotometric measurement using a NanoDrop 2000 system (Thermo Fisher Scientific, USA). Total RNA concentrations were approximately 500–2000 ng/µL, and the A260/A280 ratios were within the acceptable range (1.9–2.1). Reverse transcription was performed to generate first-strand cDNA using the PrimeScript™ RT-PCR Kit (Takara, Tokyo, Japan). For qPCR analysis, the resulting cDNA templates were normalized by a uniform 20-fold dilution prior to amplification. Quantification of gene transcripts was carried out using real-time PCR on a LightCycler 480 platform (Roche Diagnostics, Rotkreuz, Switzerland). Each amplification reaction was prepared in a 10 μL volume comprising SYBR^®^ Green Pro Taq HS Premix II (2×), template cDNA, specific primer pairs, and nuclease-free water. The PCR cycling conditions included an initial enzyme activation step at 95 °C for 2 min, followed by 40 amplification cycles consisting of denaturation at 95 °C for 15 s, primer annealing at 58 °C for 15 s, and elongation at 72 °C for 20 s. β-actin was selected as the reference gene for normalization, and relative transcriptional changes were calculated using the comparative CT (2^−ΔΔCT^) approach [23]. Detailed primer information is provided in Table 2.

### 2.6. 16S rRNA Gene Sequencing Analysis

Microbial genomic DNA was obtained from intestinal samples using a commercial Stool/Soil DNA isolation kit (VAMNE, Vazyme, Nanjing, China). To achieve species-level resolution of bacterial communities, near-complete 16S rRNA gene fragments encompassing the V1-V9 hypervariable regions were selectively amplified with the universal primers 27F and 1492R. The resulting PCR products were purified and utilized for long-read sequencing library construction. Sequencing was performed on a PacBio Sequel II platform by Beijing Biomarker Technologies Co., Ltd. (Beijing, China). Raw polymerase reads were processed to generate circular consensus sequences (CCSs) using SMRT Link software (SMRT Link v25.3). High-quality CCS reads were then separated according to sample-specific barcodes, transformed into FASTQ files, and subjected to downstream microbial community analysis using the BMKCloud bioinformatics pipeline.

### 2.7. Calculation Formula

Survival rate (SR, %): $SR,\%=\frac{N_{t}}{N_{0}}\times 100$
Weight gain rate (WGR, %): $WGR=\frac{W_{t}-W_{0}}{W_{0}}\times 100$
Feed conversion ratio (FCR): $FCR=\frac{F}{W_{t}-W_{0}}$
Specific growth rate (SGR, %/day): $SGR=\frac{lnW_{t}-lnW_{0}}{D}\times 100$
Feed intake (FI, g/shrimp): $FI=\frac{F}{N_{0}}$
Condition factor (CF, %): $CF=\frac{W_{t}}{L^{3}}\times 100$
Hepatosomatic index (HSI, %): $HSI=\frac{W_{h}}{W_{t}}\times 100$

Where *N*_0_ and *N_t_* represent the initial and final numbers of shrimp, respectively; *W*_0_ and *W_t_* denote the initial and final mean body weights (g); *F* indicates the total dry feed intake (g); *D* is the feeding duration (days); *L* represents body length (cm); and *W_h_* refers to hepatopancreas weight (g).

### 2.8. Statistical Analysis

Data analysis was conducted using one-way ANOVA in SPSS (v22.0), followed by Duncan’s test for post hoc comparisons. Statistical significance was accepted at *p* < 0.05, and values are reported as mean ± SEM.

## 3. Results

### 3.1. Growth Performance

As shown in Table 3, shrimp fed the CON and CBPH diets exhibited significantly greater FBW, WGR, and SGR compared with those fed the LFM diet (*p* < 0.05). In addition, dietary supplementation with CBPH significantly increased FI while reducing FCR relative to the LFM group (*p* < 0.05). No significant differences were observed among dietary treatments with respect to SR, CF, and HSI (*p* > 0.05).

### 3.2. Whole Body Composition

Whole-body composition is given in Table 4, there were no significant differences in moisture and ash contents among the dietary groups (*p* > 0.05). However, both crude protein and crude lipid levels were found to be significantly higher in the CON and CBPH groups when compared to the LFM group (*p* < 0.05).

### 3.3. Hemolymph Serum Biochemical Parameters

Table 5 shows the hemolymph serum biochemical indexes of Pacific white shrimp. TP increased significantly in CON and CBPH compared with group LFM (*p* < 0.05), but TG remained comparable across diets (*p* > 0.05). GLU concentration was significantly lower in the CBPH group than in other groups (*p* < 0.05). Notably, LFM diet produced the highest AST and ALT activities, and the inclusion of CBPH markedly lowered these activities (*p* < 0.05).

### 3.4. Appetite Gene mRNA Expression in the Intestine

**Figure 1:**
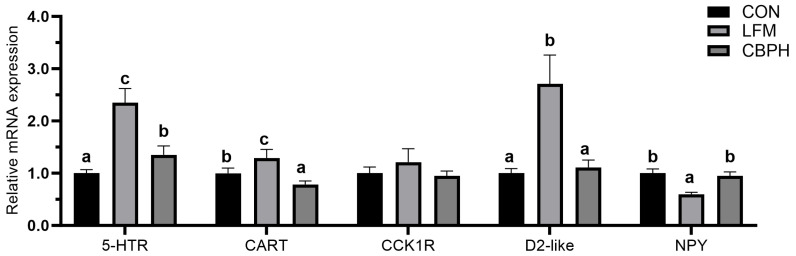
Effects of low-fishmeal dietary supplementary CBPH on appetite-related gene expression of Pacific white shrimp. Mean ± SEM is shown with upright bars (*n* = 4). The vertical bars show the average values plus or minus the standard error of the mean (for four samples). Bars with different letters show significant differences (*p* < 0.05).

Figure 1 illustrates the relative mRNA expression levels of appetite-related genes in the intestine of Pacific white shrimp. The expression of *5*-HTR, CART and D2-like was significantly higher in the LFM group than that in CON group (*p* < 0.05), whereas CBPH supplementation significantly lowered 5-HTR, CART and D2-like expression compared with the LFM group. No significant differences were detected in CCK1R expression among the three experimental groups (*p* < 0.05). NPY expression was notably reduced in the LFM group compared with the CON group (*p* < 0.05), whereas CBPH supplementation significantly increased NPY expression relative to the LFM group.

### 3.5. Intestinal Microbiota

#### 3.5.1. Intestinal Microbial Alpha Diversity

Table 6 shows the intestinal microbial alpha diversity index. The CBPH group showed higher ACE and Chao1 than the LFM group (*p* < 0.05), while the CON group did not differ significantly from either LFM or CBPH (*p* > 0.05). Shannon and Simpson were higher in CON than in LFM and CBPH, with no significant difference between LFM and CBPH (*p* > 0.05).

#### 3.5.2. Intestinal Microbial Composition and OTU Distribution

**Figure 2:**
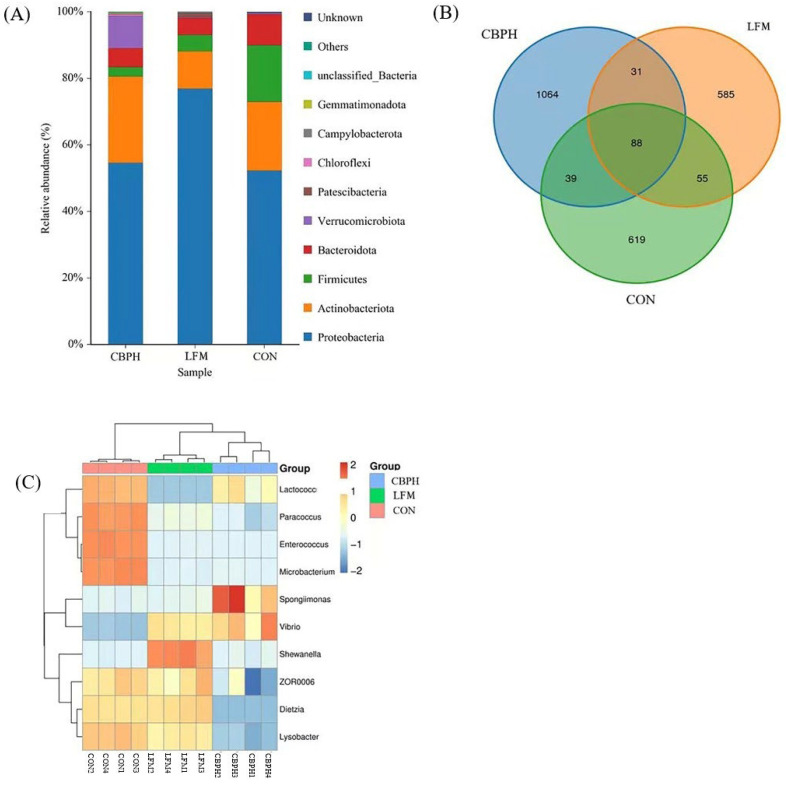
Effects of low-fishmeal dietary supplementary CBPH on the bacterial species composition of the intestinal microbiota community of Pacific white shrimp. (**A**) Taxonomic distribution; (**B**) Venn diagram; (**C**) Heat map.

As presented in Figure 2A, the intestinal microbiota of all treatment groups was primarily composed of *Proteobacteria*, *Actinobacteriota*, *Firmicutes*, and *Bacteroidota* at the phylum level. Among the groups, the LFM group exhibited the highest relative abundance of *Proteobacteria* (76.90%) and the lowest proportion of *Actinobacteriota* (11.25%). *Verrucomicrobiota* was notably more abundant in the CBPH group (9.76%) than in the CON (0.38%) and LFM (0.21%) groups. As illustrated in Figure 2B, 88 OTUs overlapped among the three groups. The CBPH group displayed the greatest number of unique OTUs (1064), followed by the CON group (619) and the LFM group (585). Regarding the heat map in Figure 2C, *Enterococcus* and *Microbacterium* were the predominant genera in the CON group, *Shewanella* was most abundant in the LFM group, and *Spongiimonas* was dominant in the CBPH group. Unclassified or provisional taxa (e.g., ZOR lineages) were retained as reported by the reference database.

## 4. Discussion

The reduction in dietary fishmeal markedly compromised growth performance and feed efficiency in Pacific white shrimp, reflecting nutritional and palatability constraints inherent to low-fishmeal formulations. In contrast, supplementation with crayfish by-product protein hydrolysate substantially mitigated these adverse effects, as demonstrated by increased feed intake, improved feed conversion, and growth outcomes comparable to those of shrimp fed the commercial control diet. These observations indicate that CBPH can functionally compensate for reduced fishmeal inclusion by enhancing diet palatability and optimizing nutrient utilization. Similar growth-promoting effects of enzymatically derived protein hydrolysates have been documented in juvenile rainbow trout, European seabass, and Pacific white shrimp, where hydrolyzed fish by-products, feather-based hydrolysates, or aquaculture residues were incorporated into low-fishmeal diets [24,25,26]. Consistently, the present study showed that CBPH supplementation significantly elevated feed intake while reducing feed conversion ratio relative to the unsupplemented low-fishmeal diet, further supporting its role as a functional additive for stabilizing shrimp performance under fishmeal-restricted conditions.

Condition factor (CF) and hepatosomatic index (HSI) are commonly used morphological indicators of general health status and nutritional condition in aquatic species [27]. In the present experiment, neither CF nor HSI differed significantly among dietary treatments, suggesting that CBPH inclusion did not adversely affect somatic growth patterns or hepatopancreatic development. Comparable results have been reported in juvenile rainbow trout fed enzymatically hydrolyzed fish by-products [28] and in genetically improved tilapia receiving enzymatic chicken liver [29], indicating that dietary protein hydrolysates generally exert neutral effects on these morphological indices. Whole-body proximate composition provides additional insight into nutrient deposition and metabolic status [30,31]. In this study, shrimp fed the CBPH-supplemented diet exhibited significantly higher crude protein and lipid contents than those fed the low-fishmeal diet alone, implying enhanced assimilation and retention of dietary nutrients. These compositional changes are consistent with the improved growth performance observed under CBPH supplementation.

Hemolymph biochemical parameters are widely applied as indicators of metabolic condition and physiological homeostasis in aquatic animals [32,33]. Elevated total protein levels are often associated with enhanced metabolic activity and improved health status [34]. In agreement with this concept, shrimp receiving the CBPH-supplemented diet displayed significantly higher hemolymph total protein concentrations than those fed the low-fishmeal diet, consistent with previous observations in fish fed hydrolysate-enriched diets [35]. Triglyceride concentrations remained unaffected by dietary treatment, indicating that lipid transport and storage were not markedly altered. In contrast, shrimp fed the low-fishmeal diet exhibited pronounced increases in AST and ALT activities, enzymes that are sensitive markers of hepatopancreatic and tissue metabolic stress [36,37]. The inclusion of CBPH effectively reduced the activities of both enzymes, suggesting an alleviation of diet-induced metabolic burden. Similar regulatory effects of crayfish-derived protein hydrolysates on aminotransferase activities have been reported in zebrafish [21]. Collectively, these findings indicate that CBPH supplementation contributes to improved metabolic stability and hepatopancreatic health in shrimp fed low-fishmeal diets.

Appetite regulation plays a key role in mediating dietary effects on feeding behavior [38,39]. In this study, qPCR analysis revealed that shrimp fed the low-fishmeal diet exhibited significantly higher intestinal expression of 5-HTR, CART, and D2-like, which are generally associated with anorexigenic signaling and reduced feeding activity [40,41,42], suggesting suppressed feeding motivation under reduced fishmeal conditions. Similar appetite-inhibitory roles of serotonergic and dopaminergic signaling, as well as CART-mediated pathways, have been reported in crustaceans and other aquatic organisms, where these factors participate in the modulation of feeding behavior and energy balance [43]. In contrast, CBPH supplementation markedly downregulated the expression of 5-HTR, CART, and D2-like, indicating an alleviation of diet-induced anorexigenic regulation. Meanwhile, no significant differences were observed in CCK1R expression among dietary treatments, suggesting that CCK-mediated satiety signaling may be less responsive to fishmeal substitution under the present experimental conditions, which is consistent with previous observations that CCK/CCK1R responses can be species- and context-dependent in aquatic animals [44]. Neuropeptide Y (NPY) is a classic orexigenic factor that promotes feed intake and promotes growth in penaeid shrimp and other aquatic species [45]. NPY expression was significantly reduced in the LFM group but was effectively upregulated following CBPH supplementation. Collectively, these transcriptional changes suggest that CBPH supplementation modulates intestinal appetite-related gene expression by suppressing anorexigenic factors while enhancing orexigenic cues, thereby providing a molecular basis for improved feeding performance in shrimp fed low-fishmeal diets.

The intestinal microbiota constitutes a critical component of the host metabolic ecosystem, influencing nutrient metabolism, immune function, and growth performance in aquatic animals [46,47]. Across all dietary treatments, the shrimp intestinal microbiota was dominated by *Proteobacteria*, *Actinobacteriota*, *Firmicutes*, and *Bacteroidota*, indicating the presence of a conserved core community structure. Nevertheless, dietary composition exerted a clear influence on microbial diversity and community complexity. Microbial diversity is widely regarded as an indicator of intestinal ecosystem stability and functional resilience, with greater diversity often associated with improved host health [48,49]. Operational taxonomic unit (OTU) richness in shrimp is known to increase with development, environmental complexity, and dietary intervention [50,51]. In the present study, CBPH supplementation increased the number of OTUs and moderately elevated alpha diversity indices relative to the low-fishmeal diet without CBPH. These microbiota changes coincided with enhanced growth performance and feed utilization, suggesting that CBPH may indirectly support shrimp health and nutritional status by promoting a more diverse and functionally robust intestinal microbial community under low-fishmeal feeding conditions. Nevertheless, this study is limited by the evaluation of a single CBPH inclusion level and the lack of direct metabolomic and functional validation of microbiota-host interactions, which warrant further investigation.

## 5. Conclusions

In conclusion, dietary supplementation with 2% crayfish by-product protein hydrolysate effectively mitigated low-fishmeal-associated reductions in feed intake, growth performance, and feed efficiency in Pacific white shrimp. These improvements were accompanied by favorable modulation of hemolymph serum metabolic biomarkers (reduced AST/ALT and glucose levels), appetite-related transcriptional responses, characterized by the downregulation of anorexigenic genes (5-HTR, CART, D2-like), upregulation of the orexigenic gene NPY and increased intestinal gut microbiota richness and composition. These results support CBPH as a sustainable functional ingredient to stabilize metabolic responses and performance in low-fishmeal shrimp feeds. Moreover, this study provides a practical strategy to improve the sustainability and circular utilization of freshwater crustacean by-products in shrimp aquafeed formulations.

## Figures and Tables

**Table 1:** Experimental diet formulation and proximate composition.

Ingredients, %	CON	LFM	CBPH
Red fish meal	20	10	10
Chicken meal	5	5	5
Soybean meal	23	27	27
Peanut meal	14	14	14
Soy protein concentrate	3	9	9
Wheat meal	24.67	24.05	22.05
Beer yeast	2	2	2
Fish oil	0	0.6	0.6
Soybean lecithin	2.5	2.5	2.5
Soybean oil	1.5	1.5	1.5
Vitamin premix ^a^	0.5	0.5	0.5
Mineral premix ^b^	0.5	0.5	0.5
Choline chloride	0.2	0.2	0.2
Cholesterol	0.05	0.05	0.05
Ca(H_2_PO_4_)_2_	1.7	1.7	1.7
Lysine	0.04	0.04	0.04
Ascorbic phosphate ester	0.1	0.1	0.1
Methionine	0.22	0.24	0.24
Threonine	0.02	0.02	0.02
Alginic acid	1	1	1
CBPH	0	0	2
Proximate composition (%, wet weight basis)			
Moisture	9.18	9.23	9.13
Crude protein	39.53	39.16	39.27
Crude lipid	7.61	7.34	7.45
Ash	9.31	9.47	9.54

^a^ Vitamin premix (per kg of mixture): vitamin A (250,000 IU), vitamin D_3_ (45,000 IU), vitamin E (3750 mg), vitamin C (7000 mg), vitamin B_1_ (500 mg), vitamin B_2_ (750 mg), pyridoxine hydrochloride (500 mg), vitamin B_12_ (1 mg), vitamin K (250 mg), folic acid (125 mg), biotin (vitamin H, 10 mg), calcium pantothenate (1250 mg), nicotinic acid (2000 mg), and inositol (2500 mg). ^b^ Mineral premix (per kg of mixture): potassium (22,500 mg), magnesium (3000 mg), zinc (4000 mg), copper (500 mg), iron (as ferrous sulfate, 200 mg), iodine (200 mg), cobalt (50 mg), selenium (10 mg), and sodium chloride (2.6 g).

**Table 2:** Sequences of primers designed for quantitative gene expression analysis.

Primers	Sequences (5′-3′)	Accession No.	Ta (°C)	Product Length (bp)
5-HTR	F: GTTGTGTCTGTTACGGGCCT	XM_070120625	58	149
	R: GCACCGTATTTCAAACCGCA			
CART	F: GTAGACGCTGAGGATGGCTC	XM_070135670	58	101
	R: GTCACCTCACTGGACACAGG			
CCK1R	F: TTCTGGTGGAACCATCACGG	XM_070141426	58	211
	R: AACATATGAAGCGTCCGGCT			
D2-like	F: TTTCCCTGTGCTTTCACCGT	XM_070131267	58	129
	R: ACACACCCGGTTTCTGTTGT			
NPY	F: AGCTTCGCAGATTCAAGCCT	XM_027366020	58	85
	R: CTGAGTCGCACGTCCTTCAT			
β-actin	F: CCACGAGACCACCTACAAC	GFRP01025709	58	142
	R: AGCGAGGGCAGTGATTTC			

5-HTR, 5-hydroxytryptamine receptor; CART, cocaine and amphetamine-regulated transcript; CCK1R, cholecystokinin receptor 1; D2-like, dopamine receptor D2-like; NPY, neuropeptide Y; β-actin, beta-actin.

**Table 3:** Growth Performance and morphological parameters of Pacific white shrimp receiving CBPH-supplemented low-fishmeal diets.

	CON	LFM	CBPH
FBW (g)	9.02 ± 0.28 ^b^	8.06 ± 0.32 ^a^	8.83 ± 0.25 ^b^
SR (%)	96.67 ± 3.85	91.67 ± 4.30	90.83 ± 3.19
FI (g)	10.71 ± 0.21 ^b^	10.06 ± 0.25 ^a^	10.69 ± 0.20 ^b^
WGR (%)	1858.74 ± 76.75 ^b^	1673.33 ± 50.70 ^a^	1782.38 ± 64.98 ^b^
SGR (%)	5.31 ± 0.07 ^b^	5.13 ± 0.05 ^a^	5.24 ± 0.06 ^b^
FCR	1.25 ± 0.02 ^a^	1.33 ± 0.03 ^b^	1.28 ± 0.02 ^a^
CF(g/cm^3^)	1.05 ± 0.02	1.03 ± 0.02	1.07 ± 0.03
HSI (%)	5.07 ± 0.11	4.94 ± 0.09	5.08 ± 0.09

Note: Results are reported as mean ± SEM (*n* = 4). Different superscript letters in the same row indicate statistically significant differences (*p* < 0.05).

**Table 4:** Whole body composition of Pacific white shrimp (wet basis).

	CON	LFM	CBPH
Moisture (%)	74.82 ± 0.58	74.91 ± 0.10	74.45 ± 0.04
Crude Protein (%)	16.98 ± 0.12 ^b^	16.36 ± 0.05 ^a^	17.00 ± 0.17 ^b^
Crude lipid (%)	1.67 ± 0.03 ^b^	1.55 ± 0.02 ^a^	1.64 ± 0.03 ^b^
Ash (%)	3.42 ± 0.15	3.37 ± 0.03	3.20 ± 0.08

Note: Results are reported as mean ± SEM (*n* = 4). Different superscript letters in the same row indicate statistically significant differences (*p* < 0.05).

**Table 5:** Hemolymph biochemical parameters of Pacific white shrimp.

	CON	LFM	CBPH
Total protein (U/mg)	65.93 ± 1.33 ^b^	61.53 ± 1.01 ^a^	68.40 ± 1.13 ^b^
Triglyceride (mmol/L)	0.92 ± 0.05	1.03 ± 0.11	1.00 ± 0.06
Glucose (mmol/L)	1.98 ± 0.25 ^ab^	2.28 ± 0.15 ^b^	1.66 ± 0.07 ^a^
AST (U/L)	236.25 ± 4.85 ^b^	303.75 ± 3.75 ^c^	209.00 ± 12.76 ^a^
ALT (U/L)	207.50 ± 5.84 ^b^	252.75 ± 3.35 ^c^	178.75 ± 2.84 ^a^

Note: Results are reported as mean ± SEM (*n* = 4). Different superscript letters in the same row indicate statistically significant differences (*p* < 0.05). AST, aspartate transaminase; ALT, alanine transaminase.

**Table 6:** Intestinal microbial alpha-diversity indices of Pacific white shrimp.

	CON	LFM	CBPH
ACE	326.52 ± 8.16 ^ab^	283.66 ± 12.52 ^a^	398.68 ± 49.55 ^b^
Chao1	332.06 ± 7.89 ^ab^	284.14 ± 12.93 ^a^	400.11 ± 50.41 ^b^
Shannon	5.87 ± 0.01 ^b^	4.62 ± 0.05 ^a^	4.63 ± 0.33 ^a^
Simpson	0.97 ± 0.01 ^b^	0.92 ± 0.01 ^a^	0.91 ± 0.01 ^a^

Note: Different superscript letters in the same row indicate statistically significant differences (*p* < 0.05).

## Data Availability

The datasets generated and/or analyzed during the current study are available from the corresponding author on reasonable request.
